# Prevalence of metastases within the hypothalamic-pituitary area in patients with brain metastases

**DOI:** 10.1186/s13014-019-1337-6

**Published:** 2019-08-27

**Authors:** Stefan Janssen, Preena Mehta, Tobias Bartscht, Sebastian M. Schmid, Fabian B. Fahlbusch, Dirk Rades

**Affiliations:** 1 Department of Radiation Oncology, University of Lübeck, Lübeck, Germany; 2 Private Practice of Radiation Oncology, Hannover, Germany; 3 Department of Haematology and Oncology, University of Lübeck, Lübeck, Germany; 4 Department of Endocrinology, University of Lübeck, Lübeck, Germany; 5 German Center for Diabetes Research (DZD), Neuherberg, Germany; 6 Department of Pediatrics and Adolescent Medicine, Friedrich-Alexander-University of Erlangen-Nürnberg, Erlangen, Germany; 7 Klinik für Strahlentherapie, Universität zu Lübeck, Ratzeburger Allee 160, Haus 40, 23562 Lübeck, Germany

**Keywords:** Brain metastases, Whole brain radiotherapy, Hormonal impairment, Hypothalamus/pituitary gland sparing, QoL

## Abstract

**Aim:**

To quantify the prevalence of brain metastases involving the hypothalamic-pituitary (HT-P) area.

**Introduction:**

Cognitive impairment and fatigue are common side effects of whole brain irradiation (WBI) comprising the quality of life (QoL) for survivors. While the former is related to radiation-induced hippocampal injury, the latter could be secondary to hormonal disbalance as a consequence of radiation of the HT-P area. Thus, sparing both regions from higher irradiation doses could reduce these sequelae.

**Methods:**

T1 contrast medium enhanced magnetic resonance imaging (MRI) scans of 865 patients with brain metastases (4,280 metastases) were reviewed. HT-P area was individually contoured with a margin of 5 mm in order to evaluate the prevalence of brain metastases in this region.

**Results:**

Involvement of the hypothalamic region was found in 26 patients (involvement rate of 3% for patients and 1% for metastases), involvement of the pituitary gland in 9 patients (1% for patients and < 1% for metastases). Binary logistical regression analysis revealed the presence of > 10 brain metastases as the only factor associated with hypothalamic involvement while no distinct factor was associated with an involvement of the pituitary gland.

**Conclusion:**

The low prevalence of metastases within the HT-P area in patients with brain metastases calls for further studies examining whether sparing of this region might improve patients QoL.

## Introduction

Brain metastases are the most common intracranial tumors and occur in approximately 10 to 30% of adult cancer patients [1]. Even in modern times of high precision radiotherapy, whole brain irradiation (WBI) is an option for patients with multiple brain metastases or as prophylactic cranial irradiation (PCI) in patients with small-cell lung cancer (SCLC). However, cognitive impairment and fatigue are common long-term side effects that relevantly comprise the quality of life (QoL) for survivors [2]. Hippocampal neural stem-cell injury due to irradiation might play a mechanistic role in memory decline [3] while affection of the hypothalamic-pituitary (HT-P) axis further promotes symptoms like fatigue [4]. Impaired HT-P axis activity is common after radiotherapy of brain tumors [5–8] as well as head-and-neck cancer [9–14]. Although the degree of irradiation damage is dose-dependent [7], relevant impairment of the HT-P axis can already occur upon low doses of ~ 18 Gy [15].

Long-term endocrine follow-up data from adult survivors of childhood and adolescent cancer [16, 17] who received WBI (mainly 24 Gy) show that the vast majority of patients developed some kind of HT-P insufficiency. The hormonal deficiency typically follows a predictable course [15] with growth-hormone (GH) secretion being the most sensitive to radiotherapy. In fact, it might be the first or only hormone deficiency apparent [16]. Clinically, radiation-induced GH deficiency in adults mimics signs and symptoms of adult GH deficiency syndrome, in particular increased fatigue and impaired QoL [18]. Thus, radiation-related endocrinopathy affects the patient not only physiologically, but also psychologically [16]. Other WBI-associated HT-P endocrinopathies include gonadotropin (follicle-stimulating hormone and luteinising hormone) deficiency, thyroid-stimulating hormone (TSH) deficiency, adrenocorticotropic hormone (ACTH) deficiency and hyperprolactinaemia [16].

As GH replacement therapy might improve the above symptoms [18] among survivors of childhood acute lymphoblastic leukemia and CNS tumors, provocative endocrine follow-up testing for isolated GH deficiency, e.g. by insulin tolerance test (ITT), is crucial to establish a robust diagnosis for the timely implementation of GH replacement therapy [17]. In this context, a priori avoidance of radiation-induced injury of the HT-P region might pose a feasible alternative for certain patients, especially in the palliative setting of brain metastases where preservation of QoL is the main objective. In accordance to a phase II study showing better memory function QoL when sparing hippocampus area during WBI [19], sparing the HT-P area during WBI may result in better endocrine and functional outcome.

To assess the practicability of HT-P sparing in patients with brain metastases detailed knowledge on the prevalence of metastatic lesions within this potential avoidance region is crucial. As scientific data is scarce to date, the present study aims to systematically assess the prevalence of HT-P metastases in patients with brain metastases.

## Methods

**Fig. 1:**
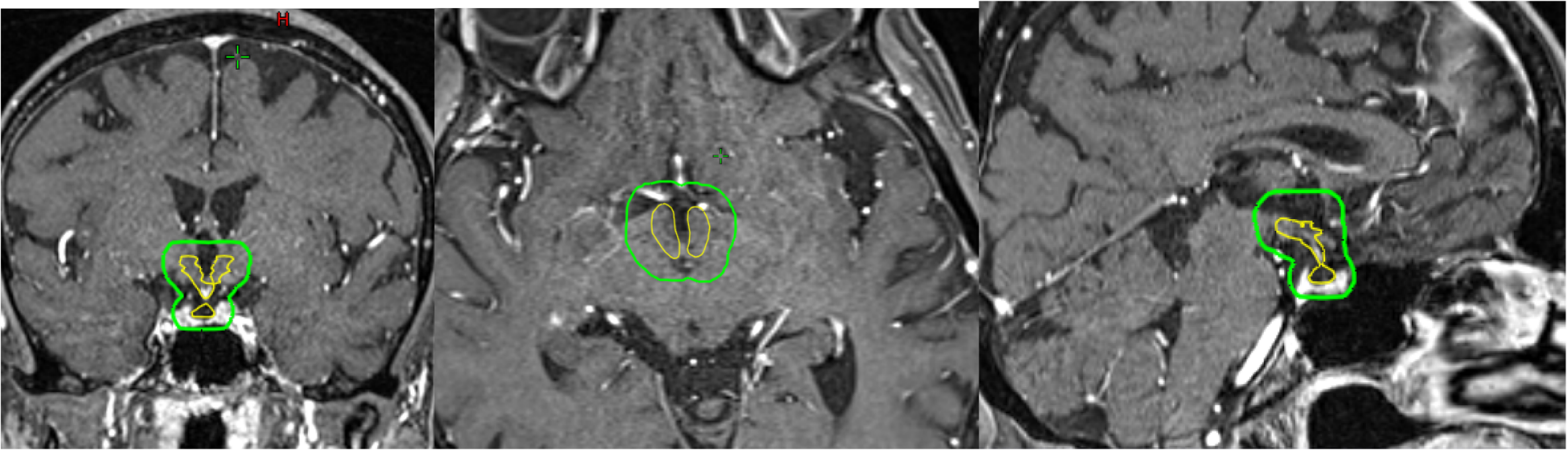
Contouring example of the HT-P axis (gadolinium enhanced T1 MRI)

Pre-treatment gadolinium-enhanced T1-weighted magnetic resonance image (MRI) datasets of 865 patients with brain metastases from the years 2014–2018 were reviewed at the University of Luebeck, Germany. The HT-P area was contoured on axial planes for each patient as described previously by others [20, 21]. In short, minimal requirements for sufficient contouring and delineation were as following: CT images (axial planes) were fused to images obtained via T1-weighted MRI images (gadolinium contrast enhanced) acquired on 1.5 T resonance scans with a slice thickness of 1.5 mm. The hypothalamus and pituitary gland (including the pituitary stalk) were contoured on T1-weighted axial MRI sequences and a margin of 5 mm was added (see Fig. 1) [20, 21].

We excluded examinations not fulfilling a minimum of standards e.g. providing only one MRI sequence, blurred sequences, slice thickness > 1.5 mm or no contrast medium.

For each patient, data regarding the primary tumor type, age at the diagnosis of brain metastases, gender, maximum size of brain metastases, and the total number of brain metastases was recorded (Table 1). In case of more than 30 brain metastases, the number was rated as ≥30. Patients with leptomeningeal disease and previous brain irradiation were excluded from analysis.

**Table 1:** Patient characteristics (NSCLC: non-small cell lung cancer, SCLC: small cell lung cancer)

*Sex*	
male	434 (50%)
female	431 (50%)
*Age*	Mean: 64 years (29–90)
< 64 years	424 (49%)
> 64 years	441 (51%)
*Primary tumor entity*	*involvement of hypothalamus and pituitary gland*
NSCLC	401 (46%) 13/401
SCLC	121 (14%) 8/121
Breast	106 (12%) 5/106
Melanoma	62 (7%) 1/62
Renal	27 (3%) 0/27
Colorectal	35 (4%) 2/35
Other	113 (13%) 6/113
Number of metastases	Mean: 5, median: 2 (range: 1–30)
Maximum size	Mean: 2 cm, median: 1.8 cm (range: 0.2 cm to 11 cm)

According to previously published analysis on hippocampal involvement, the number of metastases within 5 mm from the pituitary gland as well as from the hypothalamus was correlated with the above-mentioned variables. A binary logistical regression model using a backward step-wise approach was developed, and a two-sided *p* value of < 0.05 was considered statistically significant [22]. For the logistic regression analysis of the number of brain metastasis, we chose to analyze *n* = 1–3 vs. *n* > 10, as several randomized studies support the use of radiosurgery in 1–3 lesions [23, 24]. Moreover, some authors have shown a survival difference for this threshold [25]. Additionally, the Graded Prognostic Assessment (GPA) from the Radiation Therapy Oncology Group (RTOG) and the diagnosis-specific GPA score (ds-GPA) also differentiates between > 3 and less than three metastases [26, 27].

The protocol of this retrospective study was approved by the local ethics committee of the University of Luebeck (# 19-075A).

## Results

A total number of 4,280 brain metastases in 865 patients were identified with a mean of 5 metastases per patient. Individual maximum size of metastases ranged from 0.2 to 11 cm (median: 1.8 cm). Patients mean age was 64 years (range: 29–90 years) and the primary tumor entity was non-small cell lung cancer (NSCLC) in 46% of the patients. Patient and disease characteristics are summarized in Table 1. Involvement of the hypothalamus region (hypothalamus + 5 mm) was found in 26 patients translating to HT involvement of ~ 3% of patients and ~ 1% of all metastases. Involvement of the pituitary region (pituitary gland + 5 mm) was observed in 9 patients, i.e. 1% of all patients and < 1% of all metastases, respectively. Binary logistical regression models revealed a high number of metastases, i.e. > 10 vs. < 3 metastases as the only factor significantly associated with hypothalamic involvement (*p* = 0.0002; Table 2). In contrast, examined factors were not related to the involvement of the pituitary gland region (Table 3).

**Table 2:** Logistic regression analysis for incidence of metastases within 5 mm of the hypothalamus

Variable	Point estimate	95% Wald confidence limits	*p*-value
Age (age > 64 years vs. <=64 years)	2.019	0.845–4.824	0.1140
Sex (female vs. male)	0.820	0.335–2.009	0.6644
Tumor type (NSCLC as reference)			0.7543
SCLC	1.916	0.638–5.749	
Breast	1.427	0.327–6.219	
Melanoma/renal/colorectal	1.348	0.341–5.337	
Maximal diameter (> = 1.9 cm vs. <=1.8 cm)	2.146	0.899–5.125	0.0854
Number of brain metastases (> 10 vs. 1–3)	6.668	2.643–16.827	0.0002

**Table 3:** Logistic regression analysis for incidence of metastases within 5 mm of the pituitary gland

Variable	Point estimate	95% Wald confidence limits	*p*-value
Age (age > 64 years vs. <=64 years)	0.858	0.224–3.280	0.8238
Sex (female vs male)	1.722	0.380–7.794	0.4805
Tumor type (NSCLC as reference)			0.8121
SCLC	0.585	0.066–5.184	
Breast	1.118	0.189–6.614	
Melanoma/renal/colorectal	0.389	0.044–3.417	
Maximal diameter (> = 1.9 cm vs. <=1.8 cm)	2.108	0.518–8.576	0.2976
Number of brain metastases (> 10 vs 1–3)	2.410	0.427–13.601	0.4892

## Discussion

This retrospective analysis of 865 patients with a cumulative total of 4,280 brain metastases reveals that only ~ 4% of all metastases were located within the HT-P area. This study clearly extends our knowledge on the low prevalence of hypothalamic and pituitary region metastases.

Until now, most of current data were based on smaller studies, case reports, and reviews [28–34]. Marsh et al. reported on 155 retrospectively analyzed patients with a total of 935 brain metastases and only one case (< 1%) of pituitary involvement [29].

Metastases within the HT-P area are relatively rare and can arise from various tumors like thyroid carcinoma [28, 30, 31], melanoma [32], breast cancer [33] and prostate cancer [34]. Smaller case series could not identify a specific tumor predisposing for hypothalamic/pituitary metastases. These observations are in line with the structured analysis of our large cohort. There is no specific tumor entity correlated with HT-P involvement.

Brain metastases are common for many cancers and can be treated with surgery, high precision radiotherapy or, in case of multiple metastases, WBI. Furthermore, PCI can be applied in patients with SCLC, respectively. Although beneficial for oncological treatment, WBI is accompanied with various side effects such as neurocognitive impairment, fatigue, and endocrine disorders, all resulting in reduced QoL.

The HT-P area is highly sensible for irradiation and impairment of the endocrine hypothalamus-pituitary axes is common after radiotherapy. The somatotropic axis is most vulnerable to irradiation damage and can occur after doses as low as 18 Gy [15] or even 10 Gy in children or young adults [35]. However, endocrine disturbance can further include all other hormonal axis, i.e. the corticotropic, thyreotropic, and gonadotropic axis on the level of the hypothalamus and/or pituitary, respectively. Long term survivors of cancer with radiotherapy for head-and-neck tumors developed a dysfunction of at least one hormonal axis (46%), 24% had impairment of two axes, and 3% had a dysfunction in three axes [10]. Other studies report on hypopituitarism of at least one axis in up to 93% of patients treated with radiotherapy for nasopharynx cancer [12] and Madaschi et al. revealed clinically relevant hormone deficiency for the somatotropic (29%), corticotropic (22%), thyreotripic (14%) and gonadotropic (4%) axis in patients irradiated for extrasellar brain tumors [36]. Together, the overall prevalence of any degree of hypopituitarism is considered 25–100% in patients treated for nasopharynx cancer and 37–77% in patients with intracerebral tumors [14]. Of note, irradiation induced impairment of HP-axes appears to be a late onset sequela accompanied by a progression in severity over time but can also appear within the first year after radiotherapy [14].

All mentioned side effects after radiotherapy are dose-dependent [9, 10, 15, 37–40] with a clearly increased incidence of HT-P axes impairments at doses above 30 Gy. However, there are no data defining a clinically significant threshold [39]. Accordingly, established dose-fractionation concepts in WBI (10 × 3 Gy, 15 × 2.5 Gy or 20 × 2 Gy) are within the dose range of potential impairment.

It is tempting to speculate that avoiding irradiation of the HT-P area by HT-P sparing WBI technique might lower endocrine and neurocognitive burden of classical WBI and preserve patients QoL. Recently, the groups of Fan and Marsh, respectively, showed that sparing of the hippocampus and the HT-P axis during WBI is technically feasible [20, 41]. However, the question remains whether the oncological prognosis is acceptable when sparing HT-P area during WBI. The reported low prevalence below 4% of HT-P involvement in our large cohort of patients with brain metastases argues for further studies to justify the concept of HT-P area sparing WBI in analogy to the hippocampus sparing technique recently introduced by Gondi and colleagues [19]. On the background of a low risk of 8.6% for hippocampal metastases in 371 patients with brain metastases [22], hippocampus sparing WBI has been established in many radiation oncology centers worldwide.

Further supporting oncological safety of a HT-P area sparing WBI concept, the only predictor for involvement of the hypothalamus region was a total number of more than 10 brain metastases. Interestingly, Gondi et al. excluded patients with more than 10 brain metastases inherently from their analysis [22]. Considering the exclusion criteria of Gondi et al. and the results of our present study, we would recommend a HT-P sparing approach only in patients with less than 10 brain metastases. Although stereotactic radiosurgery (SRS) or fractionated stereotactic radiotherapy (FSRT) are increasingly applied concepts in patients with a limited number of brain metastases, there are still clinical indications for therapeutic and prophylactic WBI. Reducing potential side effects by a HT-P sparing WBI approach could improve patient’s endocrine outcome and QoL.

To our knowledge, our study involved the largest cohort regarding the assessment of the prevalence of HT-P area metastases by now. Our findings are somewhat limited by the fact that data were analyzed retrospectively and we cannot report on endocrine function in our patients. Moreover, our study did not cover radio-surgical approaches or their combination with WBI. Also, we did not examine the total volume of metastasis (as opposed to the diameter of the largest lesion) as proxy for intracranial tumor burden, which might additionally pose a potential risk factor for HT-P region involvement and decision making in HT-P sparing radiotherapy.

However, we are confident that the low prevalence of HT-P metastases seems worthy of further clinical evaluation of a sparing approach in selected patients. Although potential benefits of HT-P sparing WBI appear likely, a prospective intervention study is needed.

## Conclusion

Only 4% of brain metastases in this large study cohort of 865 patients with 4,280 metastases were located within the HT-P area. HT-P radiation-sparing might pose a treatment option for patients with a limited number of brain metastases and those without involvement of the HT-P region analogue to current hippocampus sparing approaches. As palliative therapy, WBI treatment courses should aim to improve or at least stabilize QoL. A prospective study evaluating potential endocrine and functional benefit of such a sparing approach during WBI is planned.

## Data Availability

Not applicable, entire data is shown within the manuscript / tables

## Abbreviations

ACTH: Adrenocorticotropic hormone
FSRT: Fractionated stereotactic radiotherapy
GH: Growth hormone
GPA: Graded Prognostic Assessment
HT-P: Hypothalamic-pituitary
ITT: Insulin tolerance test
MRI: Magnetic resonance imaging
NSCLC: Non-small cell lung cancer
PCI: Prophylactic cranial irradiation
QoL: Quality of life
RTOG: Radiation Therapy Oncology Group
SCLC: Small cell lung cancer
SRS: Stereotactic radiosurgery
TSH: Thyroid-stimulating hormone
WBI: Whole brain irradiation
